# Extracellular Vesicles in Herpes Viral Spread and Immune Evasion

**DOI:** 10.3389/fmicb.2018.02572

**Published:** 2018-10-25

**Authors:** Raquel Bello-Morales, José Antonio López-Guerrero

**Affiliations:** ^1^Departamento de Biología Molecular, Universidad Autónoma de Madrid, Madrid, Spain; ^2^Centro de Biología Molecular Severo Ochoa, CSIC-UAM, Madrid, Spain

**Keywords:** extracellular vesicles, microvesicles, exosomes, viral spread, immune evasion, HSV-1, autophagy

## Abstract

Extracellular vesicles (EVs) are involved in numerous processes during infections by both enveloped and non-enveloped viruses. Among them, herpes simplex virus type-1 (HSV-1) modulates secretory pathways, allowing EVs to exit infected cells. Many characteristics regarding the mechanisms of viral spread are still unidentified, and as such, secreted vesicles are promising candidates due to their role in intercellular communications during viral infection. Another relevant role for EVs is to protect virions from the action of neutralizing antibodies, thus increasing their stability within the host during hematogenous spread. Recent studies have suggested the participation of EVs in HSV-1 spread, wherein virion-containing microvesicles (MVs) released by infected cells were endocytosed by naïve cells, leading to a productive infection. This suggests that HSV-1 might use MVs to expand its tropism and evade the host immune response. In this review, we briefly describe the current knowledge about the involvement of EVs in viral infections in general, with a specific focus on recent research into their role in HSV-1 spread. Implications of the autophagic pathway in the biogenesis and secretion of EVs will also be discussed.

## Introduction

Extracellular vesicles (EVs) are a heterogeneous group of membrane vesicles, derived from endosomes or from the plasma membrane, secreted by almost all cell types belonging to the three domains of cellular life: Bacteria, Archaea, and Eukarya (Yañez-Mo et al., 2015; Sedgwick and D’Souza-Schorey, 2018; van Niel et al., 2018). EVs have been isolated from numerous biological fluids such as blood, saliva, urine, cerebrospinal fluid, amniotic fluid, ascetic fluid, breast milk, and seminal fluid (Yañez-Mo et al., 2015; Zaborowski et al., 2015; Kalra et al., 2016). Initially considered to be mostly cell debris, EVs have now emerged as key mediators of intercellular communication, and are currently associated with numerous physiological and pathological processes (Gyorgy et al., 2011; van der Pol et al., 2012; Yuana et al., 2013) such as cancer (Muralidharan-Chari et al., 2010; Barros et al., 2018; Nogues et al., 2018; Xu et al., 2018), infection (Silverman and Reiner, 2011; Lai et al., 2015; Schorey et al., 2015), inflammation and immune response (Robbins et al., 2016), and myelination and neuron-glia communication (Fruhbeis et al., 2013; Basso and Bonetto, 2016; Lopez-Leal and Court, 2016; Pusic et al., 2016; Holm et al., 2018).

Although the classification and nomenclature of EVs is complex, two major categories of EVs can be broadly established: (1) microvesicles (MVs) derived from shedding of the plasma membrane (Cocucci et al., 2009; Cocucci and Meldolesi, 2015); and (2) exosomes, vesicles released to the extracellular space upon fusion of multivesicular bodies (MVBs) with the plasma membrane (Colombo et al., 2014; Yañez-Mo et al., 2015; Maas et al., 2017). While exosomes are between 30 and 100 nm in diameter, MVs are much more heterogeneous, ranging from 100 nm to 1 μm in diameter (Raposo and Stoorvogel, 2013; Yuana et al., 2013). MVs are enriched in lipid rafts and commonly associated proteins such as flotillin-1, and expose phosphatidylserine (PS) on the outer plasma membrane leaflet (Scott et al., 1984; Del Conde et al., 2005; Wei et al., 2016). Exosomes, on the other hand, are enriched in tetraspanins (CD9, CD63 and CD81, among others), which are frequently used as exosomal markers (Andreu and Yañez-Mo, 2014) and also in endosomal markers such as ALIX and TSG101 (Kowal et al., 2016; Willms et al., 2016). Although the presence of PS exposed in exosomes has been postulated (Thery et al., 2009; Colombo et al., 2014; De Paoli et al., 2018) other studies question that exosomes expose PS just after secretion from cells (Lai R.C. et al., 2016; Skotland et al., 2017), remaining this point to be fully clarified.

Extracellular vesicles are also involved in viral infection (Meckes and Raab-Traub, 2011; Wurdinger et al., 2012; Alenquer and Amorim, 2015; Altan-Bonnet, 2016; Anderson et al., 2016), influencing viral entry, spread and immune evasion (Schorey et al., 2015; Kouwaki et al., 2017). Thus, EVs operate as an important system of intercellular communication between infected and uninfected cells (Meckes, 2015; Raab-Traub and Dittmer, 2017). Indeed, due to their common biogenesis pathways, EVs and viruses are considered to be close relatives, and EVs secreted by infected cells can either enhance viral spread or, on the contrary, trigger an antiviral response (Nolte-’T Hoen et al., 2016). The great variability of the role of EVs during the viral life cycle is evident, as they can produce such opposite effects as the blockage or increase of infection, as well as modulate the immune response (Wurdinger et al., 2012). Two key biological activities of EVs during viral infections are the transport of viral genomes into target cells and the intervention in cell physiology to facilitate infection (van Dongen et al., 2016).

This review will briefly describe the current knowledge about the involvement of EVs in viral infections, with a specific focus on recent research on their role in herpes simplex virus type-1 (HSV-1) spread.

### Viruses and EVs

Extracellular vesicles have been implicated in numerous processes during infections by both enveloped and non-enveloped viruses. For example, EVs play a relevant role in hepatitis C virus (HCV) spread, as virions contained in exosomes can be transported to hepatocyte-like cells, establishing a productive infection (Ramakrishnaiah et al., 2013). Likewise, the release of hepatitis A virus (HAV) enclosed in infectious EVs derived from cellular membranes (Feng et al., 2013) permits viral escape from antibodies and facilitates viral spread. Several picornaviruses can be transported in EVs; the secretion of exosomes containing enterovirus 71 (EV71) has been shown to establish a productive infection in human neuroblastoma cells (Mao et al., 2016), and coxsackievirus B can exit infected cells in MVs derived from mitophagosomes (Robinson et al., 2014; Sin et al., 2017). Gastroenteric pathogens such as noroviruses and rotaviruses have also been detected enclosed in EVs that can transfer a high inoculum to the next host, contributing, therefore, to fecal-oral transmission and enhancing the viral propagation (Santiana et al., 2018). Thus, infection with non-enveloped viruses can induce the release of MVs containing viral proteins and infectious virus, indicating novel routes of virus dissemination.

Human immunodeficiency virus 1 (HIV-1) modulates vesicle secretion through its Nef protein, which is secreted in exosomes and modifies the intracellular trafficking pathways to enhance viral infectivity (Lenassi et al., 2010; Pereira and daSilva, 2016). For retroviruses in general, the Trojan exosome hypothesis states that these viruses use the cellular exosome biogenesis pathway for formation of infectious virions and the exosome uptake pathway for a receptor-independent, Env-independent route of infection (Gould et al., 2003). According to this model, dendritic cells (DCs) capture and internalize retroviruses by endocytosis and, subsequently, some of the non-degraded virions may infect the DC-interacting CD4+ T cells, contributing to viral spread through a mechanism known as *trans*-infection. Both direct infection and *trans*-infection might coexist to a different extent depending on the maturation stage of DC subsets (Izquierdo-Useros et al., 2010).

Herpesviruses also modulate the secretion pathway of EVs to exit cells; in fact, the exosome pathway is exploited by the three subfamilies: alpha-, beta- and gamma- (Liu et al., 2017; Sadeghipour and Mathias, 2017). Thus, the secretion of exosomes by HSV-1-infected cells carrying viral RNA and stimulator of IFN genes (STING) to uninfected cells has recently been reported (Kalamvoki et al., 2014; Kalamvoki and Deschamps, 2016). The exosome secretion pathway also plays an important role in the life cycle of human herpesvirus 6 (HHV-6), whose virions are released along with intraluminal vesicles via the exosomal pathway, by fusion of the limiting membrane of MVBs—in which virus particles and exosomes are enclosed—with the plasma membrane (Mori et al., 2008). A similar role for MVBs in the release of human cytomegalovirus (HCMV) has been also suggested (Fraile-Ramos et al., 2007; Schauflinger et al., 2011). The human gamma-herpesviruses Kaposi’s sarcoma-associated herpesvirus (KSHV) and Epstein–Barr virus (EBV) also alter the protein content of exosomes, probably to modulate the tumor microenvironment, enhance viral efficiency and promote tumorigenesis (Vazirabadi et al., 2003; Meckes et al., 2010, 2013). In addition, exosomes can also transfer functional miRNAs from EBV-infected cells to subcellular sites of gene repression in uninfected recipient cells (Pegtel et al., 2010).

### HSV-1: A Brief Overview

Herpes simplex virus type-1 is a highly prevalent neurotropic human pathogen belonging to the alphaherpesvirus subfamily that can infect neurons and establish latency in these cells (Roizman et al., 2011; Miranda-Saksena et al., 2018). HSV-1 causes oral, labial and, occasionally facial lesions, and is an increasingly important cause of sexually transmitted genital herpes (Horowitz et al., 2010; Bernstein et al., 2013), producing a significant percentage of cases (Wald and Corey, 2007). This virus may also cause serious pathologies such as encephalitis or keratoconjunctivitis (Whitley, 2006). HSV-1 infects epithelial cells and subsequently travels to neurons, establishing latent infection in the trigeminal ganglia.

Herpes simplex virus type-1 has the ability to infect many different host and cell types (Karasneh and Shukla, 2011), using several different receptors and pathways. This virus can enter cells by fusion of the viral envelope with the plasma membrane, a pH-independent process, or by endocytosis, which can either be low pH-dependent or low pH-independent (Reske et al., 2007; Heldwein and Krummenacher, 2008; Akhtar and Shukla, 2009; Agelidis and Shukla, 2015; Nicola, 2016). Regardless of the pathway, HSV glycoproteins such as the receptor-binding glycoprotein D (gD), the fusion modulator complex gH/gL and the fusion effector gB are essential for virion entry. Maturation and egress follow four major stages: (a) capsid assembly and DNA packaging; (b) primary envelopment and de-envelopment; (c) tegumentation and secondary envelopment; (d) exocytosis of viral particles and/or cell-to-cell transmission (Owen et al., 2015). Secondary envelopment may also occur using the endocytic pathway, and in fact, it has been proposed that endocytosis from the plasma membrane into endocytic tubules represents the main source for HSV-1 envelopment (Hollinshead et al., 2012). According to this mode of envelopment, viral glycoproteins are exported to the plasma membrane via the secretory pathway and are subsequently endocytosed in endocytic tubules that are then used to wrap the viral nucleocapsid, forming the double-membraned intracellular virion (David, 2012; Hollinshead et al., 2012). This endocytic process is dynamin-dependent, and it plays a major role for transporting HSV-1 envelope proteins to intracellular sites of virus assembly (Albecka et al., 2016).

Herpes simplex virus type-1 may use several mechanisms to spread from infected to uninfected cells (Agelidis and Shukla, 2015). Several viral glycoproteins, such as the heterodimer gE/gI or glycoprotein gK, are necessary for release of virions from parent cells, and it has been recently reported that the host enzyme heparanase-1 is also required for viral release (Hadigal et al., 2015). On the other hand, HSV-1 can disseminate in human tissues by cell-to-cell spread—the direct passage of progeny virus from an infected cell to a neighboring one—, a mechanism that might be considered as an immune evasion strategy, since it protects the virus from immune surveillance (Campadelli-Fiume, 2007). However, many aspects concerning mechanisms of viral spread are still unidentified. In this context, secreted vesicles are interesting candidates to consider, because of their ability to participate in intercellular communications during viral infections.

### HSV-1 and EVs

Production of secreted vesicles by HSV-1-infected cells has been previously reported. Light particles (L-particles) (Szilagyi and Cunningham, 1991; McLauchlan and Rixon, 1992), the first to be described, are vesicles secreted by human and animal cells after infection with every alpha-herpesviruses tested (Heilingloh and Krawczyk, 2017). They are similar to virions in appearance, but lack the viral nucleocapsid and genome (Szilagyi and Cunningham, 1991) and are thus non-infectious. L-particles have been shown to facilitate HSV-1 infection by transfer of viral proteins and cellular factors required for viral replication and also immune evasion (Dargan and Subak-Sharpe, 1997; Kalamvoki and Deschamps, 2016). Another type of particles, pre-viral DNA replication enveloped particles (PREPs), are morphologically similar to L-particles, but differ in their relative protein composition (Dargan et al., 1995).

As mentioned above, recent studies have shown that cells infected with HSV-1 can use exosomes to export STING to uninfected cells, along with virions, viral mRNAs, microRNAs and the exosome marker protein CD9. Those results suggested that HSV-1 might limit the spread of infection from cell-to-cell in order to control its virulence and facilitate the dissemination between individuals (Kalamvoki et al., 2014; Kalamvoki and Deschamps, 2016; Deschamps and Kalamvoki, 2018). On the other hand, previous studies carried out by our group indicated that Rab27a, a small GTPase implicated in exosomes secretion (Ostrowski et al., 2010), plays an important role in HSV-1 infection of oligodendrocytic cells (Bello-Morales et al., 2012). In fact, our results showed a drastic reduction not only in viral production, but also in plaque size of Rab27a-silenced cells infected with HSV-1, suggesting that Rab27a depletion might be affecting viral egress. In addition, this GTPase seems to affect the viral assembly of other viruses, such as HIV-1 (Gerber et al., 2015) and HCMV (Fraile-Ramos et al., 2010).

More recent findings (Bello-Morales et al., 2018) have suggested the participation of MVs in HSV-1 spread. Our study described the features of MVs released by the human oligodendroglial (HOG) cell line infected with HSV-1 and their participation in the viral cycle, indicating for the first time that MVs released by HSV-1-infected cells contained virions, were endocytosed by naïve cells, and led to a productive infection. This suggests that HSV-1 spread might use MVs to expand its tropism and possibly evade the host immune response (Bello-Morales et al., 2018).

### EVs in Viral Immune Evasion

The release of virus from cells mediated by EVs plays a relevant role in viral spread and pathogenesis, and may help to expand the natural tropism of viruses to include target cells which lack canonical viral receptors. On the other hand, EVs may also transfer virus-encoded proteins and nucleic acids independently of the viral particles. For instance, both simian immunodeficiency virus (SIV) (McNamara et al., 2018) and HIV-1 (Lenassi et al., 2010; Pereira and daSilva, 2016; Puzar Dominkus et al., 2017) induce the release of Nef protein in exosomes; EBV LMP1 is also secreted in EVs (Meckes et al., 2010; Nkosi et al., 2018) and functional exosomes containing miRNAs have been detected in human clinical samples and mouse models of KSHV-associated malignancies (Chugh et al., 2013).

However, EVs also have an equally important role: to protect virions against the action of neutralizing antibodies and thus increasing their stability within the host during hematogenous spread. In this sense, acquisition of an envelope can provide resistance to neutralizing antibodies, and therefore reinforce viral spread (Sin et al., 2015), since neutralizing antibodies would be ineffective against virions protected within vesicles (Robinson et al., 2014). Systemic circulation of viruses enclosed in EVs would allow them to modulate host cells without exposing their proteins or progeny virions to the immune system (Raab-Traub and Dittmer, 2017). This is especially relevant for non-enveloped viruses; thus, acquisition of an envelope helps coxsackievirus to evade the immune system, permitting an efficient non-lytic viral spread (Sin et al., 2015). But enveloped viruses may also benefit from MV-mediated spread, as HCV transmission is enhanced by exosomes (Masciopinto et al., 2004; Bukong et al., 2014), and HCV-related exosomes seem to be involved in immune escape (Shen et al., 2017). In fact, hepatic exosomes—partially resistant to antibody neutralization—can transport HCV to cells and establish a productive infection (Ramakrishnaiah et al., 2013). Likewise, HAV exploits EVs as vehicles to escape antibody-mediated neutralization (Feng et al., 2013).

Regarding HSV-1, it is accepted that this virus influences the EV pathway to enhance infection and/or evade the immune system. For example, it has been demonstrated that HSV-1 may manipulate the MHC class II processing pathway by altering the endosomal sorting and trafficking of HLA-DR, hijacking these molecules from normal transport pathways to the cell surface and diverting them into the exosome pathway (Temme et al., 2010). In addition, and as already mentioned, functional HSV-1 proteins can be transferred to uninfected cells via L-particles, a process that suggests a viral immune escape strategy (Heilingloh et al., 2015). Likewise, the results obtained in our laboratory have shown that infection of Chinese hamster ovary (CHO) cells with virus-containing MVs was not completely neutralized by anti-HSV-1 antibodies, suggesting that they shield the virus (Bello-Morales et al., 2018).

### EVs and the Autophagic Pathway

Conventional autophagy is a eukaryotic degradative pathway in which cytoplasmic components are sequestered in autophagosomes that finally will fuse with the lysosome, degrading its contents by lysosomal hydrolases (Nakatogawa et al., 2009; Ohsumi, 2014). Autophagy plays an essential role in several physiological and pathological processes, such as xenophagy, the removal of intracellular pathogens including viruses (Wirawan et al., 2012). Autophagy significantly restrains HSV-1 infection in various cell types (Yakoub and Shukla, 2015), though some viruses have acquired the ability to modulate autophagy for their own benefit (Choi et al., 2018). In this way, HSV-1 and HIV interact with Beclin-1, thereby inhibiting autophagosome maturation (Orvedahl et al., 2007; Kyei et al., 2009).

Autophagy, however, may also have a non-degradative role: in secretory autophagy, a newly discovered pathway, autophagosomes fuse with the plasma membrane instead of lysosomes, releasing vesicles enclosing cytoplasmic cargo to the extracellular environment (Rabouille et al., 2012; Jiang et al., 2013; Zhang and Schekman, 2013; Ponpuak et al., 2015).

Many other studies have shown that viruses may use autophagic Atg proteins for morphogenesis and viral egress (Munz, 2017), for example EBV (Lee and Sugden, 2008; Granato et al., 2014; Fotheringham and Raab-Traub, 2015; Hurwitz et al., 2018), varicella zoster virus (VZV) (Buckingham et al., 2016; Grose et al., 2016) or picornaviruses (Jackson et al., 2005; Taylor and Kirkegaard, 2008; Klein and Jackson, 2011; Robinson et al., 2014; Mutsafi and Altan-Bonnet, 2018). Coxsackievirus B, for instance, may use the autophagic pathway to exit cells enclosed in LC3-II-positive shedding MVs (Robinson et al., 2014), a process similar to the autophagosome-mediated exit without lysis (AWOL) observed in poliovirus (Taylor et al., 2009; Lai J.K. et al., 2016). According to this model, fusion between autophagosomes and endosomes generates amphisomes, LC3-II-positive vesicles enclosing viral particles which can fuse with the plasma membrane to secrete virions (Lai J.K. et al., 2016). Remarkably, due to the complexity of their replication cycles, viruses may exert a dual role on this kind of process: on one hand, subversion of autophagy in infected cells and on the other, induction of hyper-autophagy in bystander cells (Killian, 2012).

**FIGURE 1:**
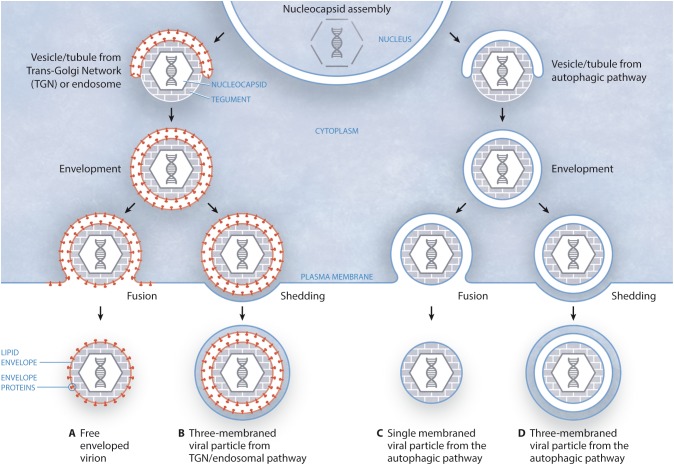
Models of biogenesis and secretion of MV-enclosed HSV-1 virions. **(A)** The canonical egress of HSV-1 entails the fusion of a two-membraned viral particle with the plasma membrane, giving rise to an extracellular free enveloped virion. In this model, the viral envelope is derived from the TGN/endosomes. **(B)** Alternatively, this structure might exit the cell after shedding of the plasma membrane, resulting in a three-membraned viral particle, which would correspond to an enveloped virion enclosed within a shedding MV. **(C)** According to this model, vesicles/tubules originating from the autophagic pathway wrap around the nucleocapsid, giving rise to a viral particle surrounded by a double membrane. Then, this structure would reach the plasma membrane and, after fusion, a viral particle surrounded by a single membrane would exit the cell. **(D)** In an alternative model, the two-membraned viral particle might exit the cell not by fusion, but by shedding of the plasma membrane, giving rise to a three-membraned viral particle.

In a recent study carried out by our group (Bello-Morales et al., 2018) we have shown the presence of virions in MVs released by infected cells. Although the exact process of HSV-1 targeting to MVs remains to be fully unraveled, we suggested that autophagy may be involved in that process, since MVs isolated from HSV-1-infected cells were positive for the autophagy marker LC3-II. Therefore, these results suggest a role for the autophagic pathway in MV-mediated HSV-1 spread, although more data is necessary to confirm that crucial point. On the other hand, this particular study suggested different models of biogenesis and secretion of MV-associated HSV-1 depending on the cell type. According to the canonical model of HSV-1 egress, the nucleocapsid is wrapped in vesicles/tubules from trans-Golgi network (TGN)/endosomes, giving rise to an enveloped virion surrounded by a double membrane that, after fusion with the plasma membrane, gives rise to an extracellular free enveloped virion (Figure 1A). However, the egress of this structure by membrane shedding cannot be excluded, which would produce a three-membraned viral particle corresponding to an enveloped virion enclosed within a shedding MV (Figure 1B). Such triple-membraned virions have been observed upon infection of Mewo cells (Bello-Morales et al., 2018); however, nucleocapsids might also obtain their envelopes from the autophagic pathway. In this way, vesicles/tubules from the autophagic pathway would cover the nucleocapsid, giving rise to a viral particle surrounded by a double membrane. Then, this structure would reach the plasma membrane and, after fusion, a viral particle surrounded by a single membrane would exit the cell (Figure 1C). This type of structure has been observed upon infection of HOG and Hela cells (Bello-Morales et al., 2018). Alternatively, double-membraned particles might exit the cell not by fusion, but by shedding of the plasma membrane, originating a three-membraned viral particle (Figure 1D). Further work will have to unveil whether viral glycoproteins are targeted to vesicles/tubules from autophagic pathway (Figures 1C,D) before wrapping of nucleocapsids. Finally, our results highlight the relevance of the cellular model when attempting the study of viral modulation of autophagy and, as different cell types may give different autophagy responses, it can be difficult to directly compare different virus-cell systems, especially when studying viruses that infect more than one cell type (Lin et al., 2010).

## Conclusion

Several herpesviruses may modulate the secretory pathway of EVs in order to enhance viral egress or evade the immune response. EVs may also increase viral stability during hematogenous spread by protecting virus from exposure to neutralizing antibodies. Recent studies have shown that MVs released by HSV-1 infected cells contained virions which were endocytosed by naïve cells, leading to a productive infection, suggesting that the virus uses EVs to expand its tropism and possibly evade the host immune response. In addition, growing evidence is demonstrating the importance of autophagy in viral infections, which several viruses use for morphogenesis and viral egress. We suggest that HSV-1 might use MVs for viral spread and propose different models of biogenesis and secretion of MVs-associated HSV-1 depending on the cell type.

## Author Contributions

Both authors conceived and wrote the manuscript.

## Conflict of Interest Statement

The authors declare that the research was conducted in the absence of any commercial or financial relationships that could be construed as a potential conflict of interest.
